# Antioxidant Delivery Pathways in the Anterior Eye

**DOI:** 10.1155/2013/207250

**Published:** 2013-09-26

**Authors:** Ankita Umapathy, Paul Donaldson, Julie Lim

**Affiliations:** ^1^Department of Optometry and Vision Science, University of Auckland, Auckland 1023, New Zealand; ^2^New Zealand National Eye Centre, University of Auckland, Auckland 1023, New Zealand; ^3^School of Medical Sciences, University of Auckland, Auckland 1023, New Zealand

## Abstract

Tissues in the anterior segment of the eye are particular vulnerable to oxidative stress. To minimise oxidative stress, ocular tissues utilise a range of antioxidant defence systems which include nonenzymatic and enzymatic antioxidants in combination with repair and chaperone systems. However, as we age our antioxidant defence systems are overwhelmed resulting in increased oxidative stress and damage to tissues of the eye and the onset of various ocular pathologies such as corneal opacities, lens cataracts, and glaucoma. While it is well established that nonenzymatic antioxidants such as ascorbic acid and glutathione are important in protecting ocular tissues from oxidative stress, less is known about the delivery mechanisms used to accumulate these endogenous antioxidants in the different tissues of the eye. This review aims to summarise what is currently known about the antioxidant transport pathways in the anterior eye and how a deeper understanding of these transport systems with respect to ocular physiology could be used to increase antioxidant levels and delay the onset of eye diseases.

## 1. Introduction

We are constantly exposed to oxidative stress through our environment, by products of metabolism and lifestyle factors. The positioning of tissues in the anterior segment of the eye makes them particularly susceptible to oxidative stress. Oxidative stress stimulates the production of unstable and highly reactive oxygen species (ROS) that are responsible for cellular damage. To combat the ubiquitous presence of ROS, ocular tissues have evolved diverse antioxidant defence systems to prevent permanent and lasting ROS-mediated tissue damage. However, as we age, prooxidants overwhelm the antioxidant defences resulting in oxidative stress and damage to tissues of the eye associated with the development of ocular pathologies such as corneal opacities, cataracts, and glaucoma. It is therefore no surprise that the therapeutic potential of natural and nutraceutical antioxidants in treating eye diseases is widely explored within vision research [1]. However, epidemiological studies into the use of such supplements have produced mixed results (reviewed by [2]). The uncertainty associated with the efficacy of antioxidant supplements is compounded by a lack of fundamental knowledge on the molecular pathways via which antioxidants accumulate in these tissues as well as a lack of understanding of basic physiological principles that underpin ocular function. Understanding and appreciating the integrated nature of antioxidant delivery and antioxidant metabolism pathways of the different tissues of the anterior eye with respect to ocular physiology are required for the design of antioxidant targeted therapies that will be effective in delaying the onset of corneal oedema, cataracts, and glaucoma—conditions for which preventive approaches do not currently exist.

## 2. Anatomy and Physiology of Ocular Tissues in the Anterior Segment

The cornea is the primary barrier between the environment and ocular tissues. It is responsible for ~75% of the total refractive power of the eye, a function owing to its transparency and unique curvature. The outermost layer of the cornea is the epithelium and is constantly exposed to mechanical stress, microbial invasion, external chemical insults, and UV radiation. The middle stromal layer accounts for nearly 90% of corneal thickness and plays a key role in maintaining corneal transparency, strength, and curvature [3–5]. It consists of nerve fibres and keratocytes which produce and maintain components of the extracellular matrix such as collagen fibres and hydrated proteoglycans. The precise arrangement and concentration of components within the stroma not only bestow incredible tensile strength to the cornea but also limit light scattering. Any irregularities in this arrangement can lead to stromal oedema and loss of transparency. The endothelium is a monolayer of squamous hexagonal cells and directly interfaces with the aqueous humour. A primary function of the corneal endothelium is to maintain stromal hydration by use of endothelial pumps that actively transport ions across the membrane to generate an osmotic gradient that draws fluid away from the stroma and into the aqueous humour. The endothelium is extremely susceptible to injury, inflicted either by mechanical trauma during intraocular surgery, inflammatory responses, or an increase in intraocular pressure [5]. Unfortunately, it does not have the capacity for regeneration, and when cells are lost or lose functionality, they cannot be mitotically replaced resulting in stromal oedema. 

The primary function of the lens is to focus light onto the retina. To achieve this, the lens is composed of an ordered arrangement of crystalline fibre cells which are derived from equatorial epithelial cells which exit the cell cycle and embark upon a differentiation process that produces extensive cellular elongation, the loss of cellular organelles and nuclei, and the expression of fibre-specific proteins [6]. Since this process continues throughout life, a gradient of fibre cells at different stages of differentiation is established around an internalised nucleus of mature, anucleate fibre cells [7]. Because there is little protein turnover in the lens nucleus, proteins in this region are particularly susceptible to oxidative damage. Since the lens is avascular, it has been hypothesised that a specialised lens microcirculation system operates to deliver nutrients and antioxidants and to remove waste products [8, 9]. The working model suggests that ionic currents, carried primarily by Na^+^, enter the lens predominantly via both poles along the extracellular spaces between fibre cells. The current then propagates across fibre cell membranes and flows towards the lens surface via an intracellular pathway mediated by gap junctions which connect all fibre cells. The influx of ions is accompanied by the convection of water, oxygen, and solutes to the deeper-lying fibre cells, while the efflux of ions facilitates the removal of fibre cell waste products. Consistent with this model, ion channels and transporters have been found in distinct fibre cell membrane domains that allow the uptake and export of solutes [7]. Regional distribution of connexin protein isoforms, which comprise gap junctions, has also been noted and may cater to the metabolic needs of specific lens regions [10]. It is theorised that disruption or inadequate functioning of the circulatory system, especially pertaining to delivery of antioxidants, could lead to cataract formation [11].

**Figure 1:**
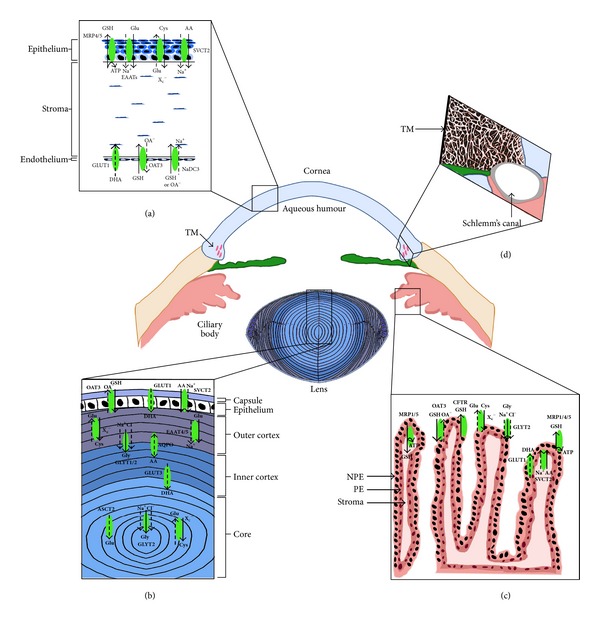
Distribution of ascorbic acid and GSH transporters in the anterior eye. All tissues appear to express similar transport mechanisms for accumulating ascorbic acid (AA) or DHA and GSH. (a) In the corneal epithelium, AA is directly accumulated from the tear fluid by SVCT2, while in the corneal endothelium, the oxidised form of AA, DHA, is taken up from the aqueous via GLUT1. In the corneal epithelium, transport of GSH precursor amino acids such as glutamate (EAATs) and cystine (X_c_^−^) have been identified as well as GSH efflux transporters such as MRP4/5. On the other hand, GSH uptake transporters OAT3 and NaDC3 were identified in the corneal endothelium, indicating that the different layers of the cornea contain different transport mechanisms for maintaining GSH levels. (b) In the lens, uptake of AA may be mediated by SVCT2 in the epithelium and AQP0 in cortical fibre cells while uptake of DHA is likely to be mediated by GLUT1 in the epithelium and GLUT3 in cortical fibre cells. GSH precursor amino acids for glutamate (EAATs), cystine (X_c_^−^), and glycine (GLYT1/2) have been identified in the outer cortex of the lens. Interestingly, X_c_^−^, GLYT2, and ASCT2 which transport glutamate at low pH, which coincide with the acidic environment of the nucleus, were also found in the lens nucleus. Since protein synthesis does not occur in this region of the lens, it has been proposed that cystine may be accumulated and then reduced to cysteine in the lens core where it can act directly as a low molecular mass antioxidant. GSH uptake transporters (OAT3) appear to be localised predominantly to the lens epithelium. (c) In the ciliary body, SVCT2 was localised to the PE layer indicating that AA in the ciliary body is taken up from the stroma. GSH precursor amino acid transporters for cystine (X_c_^−^) and glycine (GLYT2) have been identified in the NPE layer suggesting that these amino acids can be directly accumulated from the aqueous. Similarly GSH uptake transporters (OAT3) have been identified in the NPE layer. GSH efflux transporters (MRP1/4/5) have also been identified in the PE and NPE cell layers and may be involved in the removal of GSH conjugates either into the stroma or possibly into the aqueous and then removed via the trabecular meshwork. (d) To date, identification of AA and GSH uptake transporters in the trabecular meshwork is unknown.

The main function of the trabecular meshwork (TM) is to provide a site through which aqueous humour can leave the eye [12]. The TM is a mesh consisting of connective tissue progressively acquiring greater resistance to aqueous outflow towards Schlemm's canal (Figure 1(d)). The inner layer facing the anterior chamber is known as the uveal meshwork [13] and contains intertrabecular spaces that range up to 100 *μ*m, offering negligible resistance to flow [4]. Further inward is the corneoscleral meshwork which exists as several flat sheets of trabeculae that are irregularly perforated [4, 14]. In this layer, the intertrabecular space ranges between 5 and 20 *μ*m, providing a greater resistance to aqueous outflow. The outermost portion of the TM is the cribriform or juxtacanalicular meshwork which lies immediately adjacent to the endothelial cells lining Schlemm's canal [15]. This layer consists of sheets of loosely arranged TM cells that are embedded in a dense extracellular matrix composed of many types of fine collagen fibrils and proteoglycans [13, 14]. Due to the much narrower intercellular spaces, this layer provides the most resistance to aqueous humour flow in the TM [4, 13–15]. The TM thus regulates the resistance to aqueous humour outflow which is a crucial determinant of intraocular pressure (IOP) [4, 15]. An increase in the resistance of aqueous flow through the TM and therefore impaired drainage function result in elevated IOP and primary open angle glaucoma.

A common feature of these tissues is their avascular nature and their dependence on either tears or aqueous humour for their supply of glucose, amino acids, and antioxidants. The vast literature on exogenous antioxidant supplementation and the mixed findings from epidemiological studies on ocular health are not surprising given that (1) these compounds are unable to reach their target tissue via the circulatory system and (2) transporter proteins must be expressed in the cornea and lens to specifically accumulate these exogenous molecules into ocular cells. Current research is focused on designing more appropriate systems for drug delivery to the front of the eye with a great deal of attention paid towards improving ocular drug bioavailability and towards the development of noninvasive sustained drug release for anterior segment disorder. In comparison, less is known about the molecular identity of endogenous antioxidant transporter systems in the anterior eye and how these transport systems could be utilized to enhance the availability of antioxidants to specific regions within ocular tissues. 

## 3. Antioxidant Systems of the Anterior Eye

The two key antioxidants of the eye are ascorbic acid (vitamin C) and glutathione (GSH), which are present at 1 mM [16] and 2 *μ*M [17], respectively, in the human aqueous humour. While all tissues contain high levels of these antioxidants, the corneal epithelium has the highest concentration of ascorbic acid [18] and the lens has the highest concentration of GSH, indicating that these two antioxidants are particularly important in protecting these tissues from oxidative stress. 

### 3.1. Ascorbic Acid

Many mammals have the ability to synthesise ascorbic acid* de novo*; however humans and a few other species, including Wistar rats [19], lack the enzyme L-gulonolactone oxidase necessary for ascorbic acid synthesis [20] and, therefore, rely purely on dietary intake of ascorbic acid [21]. The high concentrations of ascorbic acid in the aqueous humour, together with its ability to absorb UV light, have led to its referral as a physiological “sunscreen” [22], preventing the penetration of UV light and protecting tissues from photo-induced oxidative damage [23]. As a scavenging species, ascorbic acid is oxidised by ROS in a two-step process which detoxifies or stabilises hydroxyl and superoxide anion radicals [24]. Ascorbic acid is first converted to ascorbate free radicals, which can be recycled back to ascorbic acid by ascorbate free radical (AFR) reductase [25] and then to dehydroascorbate (DHA). DHA is then reduced to regenerate ascorbic acid pools either via glutathione-dependent enzymes or nonenzymatically using low molecular weight antioxidants such as GSH or cysteine. This ensures that low levels of DHA are maintained. In the absence of ascorbic acid-DHA cycling, DHA undergoes irreversible degradation to diketogulonic acid which is implicated in modifying and crosslinking lens proteins [26, 27]. Ascorbic acid is known to protect the reducing powers of other antioxidants such as *α*-tocopherol (vitamin E) by rescuing *α*-tocopheryl radicals in membranes [28]. As a reducing agent ascorbic acid may also act as a prooxidant by reducing metal ions, which leads to the generation of free radicals through the Fenton reaction [29]. Aside from its reducing properties, ascorbic acid is also essential for collagen synthesis and has anti-inflammatory properties, preventing extensive tissue damage in the eye [30, 31].

### 3.2. Glutathione

Glutathione can also be obtained from the diet but is primarily synthesised *de novo *from the amino acids cysteine, glutamate, and glycine via the sequential actions of *γ*-glutamylsynthetase and GSH synthetase. GSH is a powerful antioxidant with multiple functions in the eye. It protects protein thiol groups by affording protection against ROS, is necessary for the functioning of several glutathione-dependent antioxidant enzymes that neutralize ROS, and is responsible for drug detoxification [32]. GSH also mediates the recycling of DHA to ascorbic acid further increasing the antioxidant capacity of the eye [33, 34]. GSH levels are maintained by an active glutathione redox cycle that ensures a high GSH to GSSG (oxidized GSH) ratio. To achieve this, the redox cycle utilizes the reciprocal actions of two enzymes: glutathione peroxidase (GPX) whose action involves the oxidation of GSH to GSSG and glutathione reductase (GR) which converts GSSG back to GSH.

### 3.3. Enzymatic Antioxidants

In the anterior chamber of the eye, superoxide dismutase (SOD), catalase, and glutathione-related enzymes act in conjunction with each other to play a critical role in regulating the production of ROS and precluding any tissue damage. While ascorbic acid is the main antioxidant in the aqueous humour, it is not as efficient in detoxifying ROS as antioxidant enzymes such as SOD [35]. SOD activity is essential for regulating the production of superoxide anions—it catalyses the conversion of superoxide anions to H_2_O_2_ which in turn can be converted to water and oxygen by catalase [36]. Catalase activity is very important because, unlike the superoxide radical, H_2_O_2_ is an uncharged molecule that can easily cross membranes. GSH-related enzymes rely on the presence of glutathione to exert their antioxidant. Glutathione reductase (GR) is a NADPH-dependent enzyme that facilitates the conversion of oxidised glutathione (GSSG) to reduced glutathione (GSH), thus maintaining the pool of total glutathione in its reduced and active form. In concert with GR, glutathione peroxidase (GPX) uses GSH to detoxify organic hydroperoxides and hydrogen peroxide resulting in the formation of GSSG and water.

## 4. Mechanisms for Accumulating Ascorbic Acid in the Anterior Eye

The positioning of the cornea means that it is constantly exposed to high amounts of UV radiation. To scavenge ROS and protect itself from light-induced damage, the corneal epithelium contains high concentrations of ascorbic acid which far exceed concentrations found in other ocular tissues [18, 23]. The corneal epithelium most likely sources ascorbic acid from tear fluids and accumulates it via the sodium-dependent vitamin C transporter 2 (SVCT2) which has been shown to be expressed in rabbit corneal epithelial cells [37] (Figure 1(a)). To a considerably lesser extent, ascorbic acid in the corneal epithelium may also be accumulated via passive diffusion from the endothelium and stroma [38]. On the other hand, the corneal endothelium preferentially takes up DHA from the aqueous humour via the facilitative glucose transporter, GLUT1, the DHA then being immediately reduced to ascorbic acid [39] (Figure 1(a)). It has also been reported to stimulate the active transport of chloride across the endothelium [40] which has been shown to significantly increase wound healing rate [41]. Ascorbic acid has been shown to be protective to endothelial cells since addition of ascorbic acid to the irrigation solution during phacoemulsification significantly reduces the amount of endothelial cell loss [42].

In the lens, ascorbic acid has many roles including prevention of membrane lipid peroxidation and the protection of lens cation pumps [19, 43]. Accumulation of ascorbic acid in the lens (~1 mM) [44] occurs by transport of both ascorbic acid and DHA (Figure 1(b)). Although DHA in the aqueous humour constitutes only about 10% of total reduced ascorbic acid, it is DHA which appears to be preferentially transported into the lens [45]. This accumulation is mediated by facilitative glucose transporters (GLUT1/3) followed by its rapid conversion to ascorbic acid [46]. DHA is known to be toxic to the lens [47] and has been linked to the formation of senile [34, 48, 49] and diabetic cataract. In human diabetes patients and animal models of diabetes leading to cataract formation, ascorbic acid levels in the lens are decreased and DHA levels are increased [50, 51]. The lens is also capable of direct ascorbic acid uptake; however this accumulation appears to be restricted to the lens epithelium [52, 53]. In a human lens epithelial cell line, ascorbic acid uptake is mediated by SVCT2, which is also upregulated in response to oxidative stress [54]. A recent study suggests that, in addition to SVCT2 and GLUTs, the water channel AQP0 may also be involved in the transport of ascorbic acid since its expression was increased in diabetic rats and upon ascorbic acid treatment [55]. Mouse fibroblasts overexpressing AQP0 displayed increased uptake of ascorbic acid and appear to aid its export, suggesting a bidirectional channel which could maintain ascorbic acid levels in lens fibres [56].

In cultured bovine trabecular meshwork cells, physiological ascorbic acid levels have been shown to promote cell growth, glycosaminoglycan production [57], and stimulation of fibronectin, laminin, and collagen type I production which are crucial for basal lamina assembly [58, 59]. Ascorbic acid may also play a protective role in minimising oxidative stress and damage to the trabecular meshwork [60]. Such damage would affect aqueous humour outflow and increase intraocular pressure, a risk factor for the development of glaucoma. Indeed, studies analysing the ascorbic acid levels in the aqueous humour of glaucoma patients and nonglaucoma/cataract controls have shown significantly lower levels of ascorbic acid in glaucoma patients [61]. Currently, the molecular identities of transporters involved in uptake of ascorbic acid/DHA into the TM are unknown.

## 5. Mechanisms for Accumulating Glutathione in the Anterior Eye 

While overall GSH concentrations in the cornea are lower relative to ascorbic acid concentrations, GSH plays a major role in corneal defence. It is involved in maintaining the barrier function of the corneal endothelium, controlling normal hydration levels, protecting cell membrane integrity, and degrading xenobiotics agents. In other tissues, GSH levels are maintained via a combination of GSH uptake, *de novo *synthesis from its precursor amino acids, GSH regeneration from GSSG by glutathione reductase, and GSH efflux. Surprisingly, there is limited information regarding the molecular pathways involved in maintaining GSH homeostasis in the cornea. Previous work has investigated GSH uptake by measuring ^35^S-GSH in the different layers of the cornea and found that the highest concentrations of ^35^S-GSH were detected in the stroma [62]. However, the transporters responsible for this GSH uptake in the cornea remain unknown. In addition, studies also suggest that GSSG rather than GSH may be better at preserving endothelial barrier function since it was found that the barrier function of the rabbit corneal endothelium was better maintained by supplementing the perfusion solution with 0.3 mM GSSG rather than 0.6 mM GSH [63]. This suggests that the corneal endothelium can rapidly turn over endothelial GSH by glutathione reductase and that the endothelium is able to preferentially accumulate GSSG [64, 65], although it is similarly unclear what transport mechanism is involved. 

A recent study into potential pathways for uptake of precursor amino acids utilized for GSH synthesis colocalized the Na^+^-dependent glutamate transporters (EAATs) and the Na^+^-independent cystine/glutamate exchanger (X_c_^−^) in the human corneal epithelium [66] (Figure 1(a)). This was followed up by a more recent study, in which it has been shown that X_c_^−^, its substrate cysteine, and the rate limiting enzyme for GSH synthesis, *γ*-glutamylcysteine synthetase (*γ*-GCS), were also localised to the rat corneal epithelium [67]. In tissues such as the brain, these two systems have been demonstrated to work together to mediate cysteine uptake for GSH synthesis [68]. The X_c_^−^ system mediates the Na^+^-independent exchange of extracellular cystine (the oxidised form of cysteine) for intracellular glutamate, while the EAATs maintain the outwardly directed glutamate concentration gradient required for glutamate exchange with cystine [69]. Upon intracellular accumulation, cystine is rapidly reduced to cysteine where it can be incorporated into proteins and/or used in the synthesis of GSH. These findings, together with others, show that the X_c_^−^ system also operates in the cornea and that GSH synthesis is most likely restricted to the epithelial layer and not the endothelium. 

The liver and kidney are major sites of GSH uptake, synthesis, and export and can therefore offer potential clues as to the molecular pathways that may operate in the cornea to maintain GSH levels. Two members of the organic anion transporter (OAT1 and OAT3) and the sodium dicarboxylate 3 transporter (NaDC3) have been shown to be involved in GSH uptake across the renal basolateral membrane [70]. NaDC3 is a secondary active transporter which couples the uptake of GSH to that of Na^+^ ions. OAT1 and OAT3 couple the outwardly directed gradient of 2-oxoglutarate with GSH uptake. Two GSH export mechanisms have been identified in the liver—the multidrug resistance associated proteins (MRPs) 1, 2, 4, and 5 and the organic anion transporting polypeptide (Oatp) 1 and 2, although only the rat Oatps and not human OATP appear to contribute to GSH release [70–72]. The MRPs mediate efflux of organic anions such as GSH at the expense of cellular ATP while the Oatps catalyses the uptake of organic anions in exchange for GSH [73]. MRPs also mediate export of oxidised GSH (GSSG), as well as glutathione-S-conjugates and the subsequent elimination of xenobiotics [70]. 

Of the three putative GSH uptake transporters, only OAT3 and NaDC3 are expressed at the transcript and protein level in the rat cornea [67]. Subsequent immunohistochemical analysis has revealed that both OAT3 and NaDC3 colocalise to the endothelium but are absent from the epithelium (Figure 1(a)). Of the putative GSH efflux transporters only MRP4 and MRP5 were detected at the transcript and protein level, and localisation studies revealed these transporters to be present in the epithelial layers only (Figure 1(a)). These findings demonstrate the corneal epithelium and endothelium to exhibit differences in GSH uptake, synthesis, and efflux pathways. The corneal epithelium appears to be the region where GSH synthesis and GSH efflux occurs. In the endothelium, however, GSH accumulation is likely to be predominantly via direct uptake of GSH from the aqueous humour. 

Among ocular tissues, the lens has the highest concentrations of GSH (6–10 mM) [74]. In the lens, GSH synthesis is restricted to the metabolically active epithelial and cortical layers, resulting in a gradient of GSH—with the highest levels being present in the central epithelium and cortical fibre cells and lowest in the lens core [75, 76]. In the rat lens, GSH precursor amino acid transporters have been reported to be involved in the uptake of cysteine, glycine, and glutamate for GSH synthesis (Figure 1(b)). We showed that in the outer cortex X_c_^−^ and EAAT4/5 colocalize indicating that these transporters may work together to accumulate cysteine for GSH synthesis [77]. On the other hand, X_c_^−^ appears to switch its glutamate recycling partner to alanine-serine-cysteine transporter (ASCT2) which transports glutamate at low pH, which coincides with the acidic environment in the lens core [78]. Since these mature anucleate fibre cells are not capable of protein/GSH synthesis, it has been proposed that X_c_^−^ and ASCT2 may work to accumulate cysteine where it can act as a low molecular mass antioxidant in this highly compact region. The glycine transporters, GLYT1 and GLYT2, were both shown to be localised to the lens cortex indicating a role in glycine uptake in cortical fibre cells (Figure 1(b)). Interestingly, GLYT2 was also expressed in the lens core. This indicates that GLYT2 may be responsible for the accumulation of glycine in the centre of the lens where it may act as an antiglycation agent to protect against protein modifications associated with age-related nuclear cataract. Furthermore, work in the authors' laboratories has identified the GSH uptake transporters OAT3 and NaDC3 but not OAT1 in the rat lens (Figure 1(b)). Immunolocalisation experiments show that NaDC3 is localized to the cytoplasm of epithelial and cortical fibre cells while OAT3 was strongly localized to the apical-apical interface indicating OAT3 to be the most likely candidate to mediate GSH uptake in this region [79]. 

In the excised bovine trabecular meshwork, glutathione is reported at a concentration of 0.40 *μ*mol/g tissue wet weight [80]. While there is no direct evidence for glutathione involvement in regulating aqueous humour outflow, it does protect against the deleterious effects of H_2_O_2_, which is known to cause tissue damage that could compromise outflow [60, 80]. In patients that exhibit open-angle glaucoma, the glutathione content of the trabecular meshwork was found to decrease with progressive deterioration of the disease, suggesting a correlation between disease pathogenesis and glutathione content [4]. A recent study has identified the presence of MRP4 in the trabecular meshwork; however its involvement in GSH transport has not been investigated [81]. Thus, the mechanisms utilised by the trabecular meshwork to accumulate GSH remain unclear.

## 6. An Integrated View of Ocular Physiology and Antioxidant Delivery

Ocular tissues are continuously subjected to a variety of physical, chemical, and environmental insults that generate ROS and create an oxidative environment in the eye. The tissues in the anterior segment of the eye, particularly the cornea, lens, and trabecular meshwork, bear the brunt of this oxidative stress due in part to their positioning in the eye and the avascular nature of these tissues. Unsurprisingly these tissues utilise similar antioxidant defence systems (Figure 1) which aid in regulating ROS production and maintaining overall ocular health. The aqueous humour is a key source of antioxidants to tissues in the front of the eye. The lens epithelium and the corneal endothelium contain transport and/or recycling pathways for accumulating antioxidants from the aqueous humour. However, to maintain overall antioxidant balance within the anterior eye, it is likely that these tissues possess intertissue signalling pathways that enable crosstalk between individual tissues. 

The ciliary body provides the aqueous humour with antioxidants, nutrients, and amino acids. However, in contrast to ion transport and fluid transport [82], little is known about the transport pathways used by the ciliary epithelium to mediate antioxidant, nutrient, and amino acid secretion. The ciliary body is believed to actively concentrate ascorbic acid from the plasma, via SVCT2 located in the pigmented epithelial (PE) cell layer [21, 83, 84] or uptake of DHA via GLUT1 located in both the PE and NPE (nonpigmented epithelial) layers [85], followed by DHA recycling [33] and then secretion of ascorbic acid into the aqueous humour [86] (Figure 1(c)). The ciliary body also possesses antioxidant enzyme activity for catalase [87], glutathione reductase [88], and glutathione peroxidase [89, 90]. Among the anterior ocular tissues, the ciliary body has one of the highest SOD activities [91, 92] and, in addition, is thought to release GPX into the aqueous humour to aid in removal of H_2_O_2_ [93]. Recently, the contribution of the ciliary epithelium to GSH levels in the aqueous humour has been examined by mapping GSH metabolism and transport pathways in the rat ciliary body [94, 95]. Both the PE and NPE cell layers are capable of accumulating precursor amino acids for GSH synthesis, but only the NPE cells appear to be involved in the direct uptake of precursor amino acids from the stroma (Figure 1(c)). The location of GSH efflux transporters to the basolateral membrane of the NPE indicates that these cells can mediate GSH secretion into the aqueous humour [95, 96]. GSH can then be used directly by anterior tissues of the eye such as the lens and cornea to protect against oxidative stress. Collectively, these studies demonstrate the ciliary body to play an important role in providing the aqueous humour with a source of antioxidants and antioxidant enzymes that can be utilised to protect anterior ocular tissues from oxidative stress. 

## 7. Therapeutic Potential

With increasing age, a decrease in antioxidants and/or antioxidant enzyme activity occurs, leading to progressive tissue damage and ocular pathologies. A large number of studies have explored the use of antioxidants (both endogenous and exogenous) in treating eye diseases. In comparison, only a few studies have investigated the molecular processes by which antioxidants accumulate in ocular tissues. For example, the use of thiol antioxidants and their derivatives, such as cysteine, glutathione, N-acetylcysteine (NAC), and more recently N-acetylcysteine amide (NACA), has been examined in providing protection from age-related nuclear cataracts [97–102]. The use of NAC and NACA has been found to be more effective against various oxidative stress-related disorders because of their ability to permeate cell membranes and bypass transporter mediated cysteine uptake. However, whether these cysteine analogues can act directly as antioxidants in the lens nucleus is unlikely since they would be rapidly incorporated into GSH in the cortex and this would limit their diffusion-dependent delivery to the lens centre. Rather a transporter-mediated pathway such as the X_c_^−^  system is likely to be more effective in accumulating cystine/cysteine directly into nuclear fibre cells. Moving ahead, identification of other transporters used to accumulate endogenous antioxidants in ocular tissues could highlight alternative pathways that could then be targeted to increase uptake and elevate antioxidant levels.

While we have described the actions of antioxidant compounds and enzymes that directly minimize oxidative stress, we have omitted enzymatic repair systems (such as thioltransferases (TTase) and thioredoxins (Trx) [103]) found in ocular tissues for the repair of oxidatively damaged and cross-linked proteins. These have been comprehensively reviewed elsewhere [104]. Yan and colleagues demonstrated that thioredoxin and thioredoxin reductase, which participate in the repair of oxidatively damaged lens protein and enzymes in combination with the chaperone protein alpha crystallin, could revive glutathione reductase activity in human cataractous lenses [105]. 

Despite the presence of immense antioxidant capacity, with age, ocular tissues can readily succumb to ROS-mediated damage. Thus, it is important to understand the transport systems and repair pathways responsible for accumulating and maintaining these antioxidants in ocular tissues, so that more targeted therapies can be developed and made successful.

## References

[1] Richer S (2000). Antioxidants and the eye. *International Ophthalmology Clinics*.

[2] Chiu C, Taylor A (2007). Nutritional antioxidants and age-related cataract and maculopathy. *Experimental Eye Research*.

[3] Riordan-Eva P, Cunningham E (2011). *Vaughan & Asbury's General Ophthalmology*.

[4] Besharse J, Dana R, Dartt DA (2010). *Encyclopedia of the Eye*.

[5] Krachmer J, Mannis M, Holland E (2005). *Cornea: Fundamentals, Diagnosis and Management*.

[6] Bassnett S, Shi Y, Vrensen GFJM (2011). Biological glass: structural determinants of eye lens transparency. *Philosophical transactions of the Royal Society of London B*.

[7] Donaldson PJ, Lim JC, Tombran-Tink J, Barnstable CJ (2008). Membrane transporters: new roles in lens cataract. *Ocular Transporters in Ophthalmic Diseases and Drug Delivery*.

[8] Candia OA, Mathias R, Gerometta R (2012). Fluid circulation determined in the isolated bovine lens. *Investigative Ophthalmology and Visual Science*.

[9] Mathias RT, Rae JL, Baldo GJ (1997). Physiological properties of the normal lens. *Physiological Reviews*.

[10] Jacobs MD, Soeller C, Sisley AMG, Cannell MB, Donaldson PJ (2004). Gap junction processing and redistribution revealed by quantitative optical measurements of connexin46 epitopes in the lens. *Investigative Ophthalmology and Visual Science*.

[11] Donaldson P, Kistler J, Mathias RT (2001). Molecular solutions to mammalian lens transparency. *News in Physiological Sciences*.

[12] Tamm ER (2009). The trabecular meshwork outflow pathways: structural and functional aspects. *Experimental Eye Research*.

[13] Bron A, Tripathi R, Tripathi B (1997). *Wolff's Anatomy of the Eye and Orbit*.

[14] Oyster CW (1999). *The Human Eye: Structure and Function*.

[15] Llobet A, Gasull X, Gual A (2003). Understanding trabecular meshwork physiology: a key to the control of intraocular pressure?. *News in Physiological Sciences*.

[16] Reiss GR, Werness PG, Zollman PE, Brubaker RF (1986). Ascorbic acid levels in the aqueous humor of nocturnal and diurnal mammals. *Archives of Ophthalmology*.

[17] Riley MV, Meyer RF, Yates EM (1980). Glutathione in the aqueous humor of human and other species. *Investigative Ophthalmology and Visual Science*.

[18] Ringvold A, Anderssen E, Kjønniksen I (2000). Distribution of ascorbate in the anterior bovine eye. *Investigative Ophthalmology and Visual Science*.

[19] Garland DL (1991). Ascorbic acid and the eye. *The American Journal of Clinical Nutrition*.

[20] Burns JJ (1957). Missing step in man, monkey and guinea pig required for the biosynthesis of L-ascorbic acid. *Nature*.

[21] Tsukaguchi H, Tokui T, Mackenzle B (1999). A family of mammalian Na^+^-dependent L-ascorbic acid transporters. *Nature*.

[22] Ringvold A (1996). The significance of ascorbate in the aqueous humour protection against UV-A and UV-B. *Experimental Eye Research*.

[23] Brubaker RF, Bourne WM, Bachman LA, McLaren JW (2000). Ascorbic acid content of human corneal epithelium. *Investigative Ophthalmology and Visual Science*.

[24] Rose RC, Richer SP, Bode AM (1998). Ocular oxidants and antioxidant protection. *Proceedings of the Society for Experimental Biology and Medicine*.

[25] Bando M, Obazawa H (1991). Regional and subcellular distribution of ascorbate free radical reductase activity in the human lens. *Tokai Journal of Experimental and Clinical Medicine*.

[26] Linetsky M, Shipova E, Cheng R, Ortwerth BJ (2008). Glycation by ascorbic acid oxidation products leads to the aggregation of lens proteins. *Biochimica et Biophysica Acta*.

[27] Fan X, Reneker LW, Obrenovich ME (2006). Vitamin C mediates chemical aging of lens crystallins by the Maillard reaction in a humanized mouse model. *Proceedings of the National Academy of Sciences of the United States of America*.

[28] Corti A, Casini AF, Pompella A (2010). Cellular pathways for transport and efflux of ascorbate and dehydroascorbate. *Archives of Biochemistry and Biophysics*.

[29] Carr A, Frei B (1999). Does vitamin C act as a pro-oxidant under physiological conditions?. *FASEB Journal*.

[30] Barnes MJ (1975). Function of ascorbic acid in collagen metabolism. *Annals of the New York Academy of Sciences*.

[31] Williams RN, Paterson CA, Eakins KE, Bhattacherjee P (1984). Ascorbic acid inhibits the activity of polymorphonuclear leukocytes in inflamed ocular tissues. *Experimental Eye Research*.

[32] Ganea E, Harding JJ (2006). Glutathione-related enzymes and the eye. *Current Eye Research*.

[33] Bode AM, Green E, Yavarow CR (1993). Ascorbic acid regeneration by bovine iris-ciliary body. *Current Eye Research*.

[34] Sasaki H, Giblin FJ, Winkler BS, Chakrapani B, Leverenz V, Shu CC (1995). A protective role for glutathione-dependent reduction of dehydroascorbic acid in lens epithelium. *Investigative Ophthalmology and Visual Science*.

[35] Behndig A, Svensson B, Marklund SL, Karlsson K (1998). Superoxide dismutase isoenzymes in the human eye. *Investigative Ophthalmology and Visual Science*.

[36] Alfonso-Prieto M, Biarnés X, Vidossich P, Rovira C (2009). The molecular mechanism of the catalase reaction. *Journal of the American Chemical Society*.

[37] Talluri RS, Katragadda S, Pal D, Mitra AK (2006). Mechanism of L-ascorbic acid uptake by rabbit corneal epithelial cells: evidence for the involvement of sodium-dependent vitamin C transporter 2. *Current Eye Research*.

[38] Choy CKM, Benzie IFF, Cho P (2004). Is ascorbate in human tears from corneal leakage or from lacrimal secretion?. *Clinical and Experimental Optometry*.

[39] Bode AM, Vanderpool SS, Carlson EC, Meyer DA, Rose RC (1991). Ascorbic acid uptake and metabolism by corneal endothelium. *Investigative Ophthalmology and Visual Science*.

[40] McGahan MC, Bentley PJ (1982). Stimulation of transepithelial sodium and chloride transport by ascorbic acid. Induction of Na^+^ channels is inhibited by amiloride. *Biochimica et Biophysica Acta*.

[41] Reid B, Song B, McCaig CD, Zhao M (2005). Wound healing in rat cornea: the role of electric currents. *FASEB Journal*.

[42] Rubowitz A, Assia EI, Rosner M, Topaz M (2003). Antioxidant protection against corneal damage by free radicals during phacoemulsification. *Investigative Ophthalmology and Visual Science*.

[43] Varma SD, Kumar S, Richards RD (1979). Light-induced damage to ocular lens cation pump: prevention by vitamin C. *Proceedings of the National Academy of Sciences of the United States of America*.

[44] Heath H (1962). The distribution and possible functions of ascorbic acid in the eye. *Experimental Eye Research*.

[45] Kern HL, Zolot SL (1987). Transport of vitamin C in the lens. *Current Eye Research*.

[46] Merriman-Smith R, Donaldson P, Kistler J (1999). Differential expression of facilitative glucose transporters GLUT1 and GLUT3 in the lens. *Investigative Ophthalmology and Visual Science*.

[47] Bianchi J, Rose RC (1986). Dehydroascorbic acid and cell membranes: possible disruptive effects. *Toxicology*.

[48] Giblin FJ, McCready JP, Kodama T, Reddy VN (1984). A direct correlation between the levels of ascorbic acid and H_2_O_2_ in aqueous humor. *Experimental Eye Research*.

[49] Spector A (1995). Oxidative stress-induced cataract: mechanism of action. *FASEB Journal*.

[50] DiMattio J (1992). Ascorbic acid entry into cornea of rat and guinea pig. *Cornea*.

[51] Yue DK, McLennan S, Fisher E (1989). Ascorbic acid metabolism and polyol pathway in diabetes. *Diabetes*.

[52] DiMattio J (1989). Active transport of ascorbic acid into lens epithelium of the rat. *Experimental Eye Research*.

[53] DiMattio J (1989). A comparative study of ascorbic acid entry into aqueous and vitreous humors of the rat and guinea pig. *Investigative Ophthalmology and Visual Science*.

[54] Kannan R, Stolz A, Ji Q, Prasad PD, Ganapathy V (2001). Vitamin C transport in human lens epithelial cells: evidence for the presence of SVCT2. *Experimental Eye Research*.

[55] Nakazawa Y, Oka M, Bando M, Inoue T, Takehana M (2011). The role of ascorbic acid transporter in the lens of streptozotocin-induced diabetic rat. *Biomedicine and Preventive Nutrition*.

[56] Nakazawa Y, Oka M, Mitsuishi A, Bando M, Takehana M (2011). Quantitative analysis of ascorbic acid permeability of aquaporin 0 in the lens. *Biochemical and Biophysical Research Communications*.

[57] Higginbotham E, Yue BYJT, Crean E, Peace J (1988). Effects of ascorbic acid on trabecular meshwork cells in culture. *Experimental Eye Research*.

[58] Yue BYJT, Higginbotham EJ, Chang IL (1990). Ascorbic acid modulates the production of fibronectin and laminin by cells from an eye tissue—trabecular meshwork. *Experimental Cell Research*.

[59] Zhou L, Higginbotham EJ, Yue BYJT (1998). Effects of ascorbic acid on levels of fibronectin, laminin and collagen type 1 in bovine trabecular meshwork in organ culture. *Current Eye Research*.

[60] Ammar DA, Hamweyah KM, Kahook MY (2012). Antioxidants protect trabecular meshwork cells from hydrogen peroxide-induced cell death. *Translational Vision Science and Technology*.

[61] Ferreira SM, Lerner SFÁ, Brunzini R, Evelson PA, Llesuy SF (2004). Oxidative stress markers in aqueous humor of glaucoma patients. *The American Journal of Ophthalmology*.

[62] Veltman JC, Podval J, Mattern J, Hall KL, Lambert RJ, Edelhauser HF (2004). The disposition and bioavailability of 35S-GSH from 35S-GSSG in BSS PLUS in rabbit ocular tissues. *Journal of Ocular Pharmacology and Therapeutics*.

[63] Nakamura M, Nakano T, Hikida M (1994). Effects of oxidized glutathione and reduced glutathione on the barrier function of the corneal endothelium. *Cornea*.

[64] Anderson EI, Wright DD (1982). The roles of glutathione reductase and *γ*-glutamyl transpeptidase in corneal transendothelial fluid transport mediated by oxidized glutathione and glucose. *Experimental Eye Research*.

[65] Ng MC, Riley MV (1980). Relation of intracellular levels and redox state of glutathione to endothelial function in the rabbit cornea. *Experimental Eye Research*.

[66] Langford MP, Redmond P, Chanis R, Misra RP, Redens TB (2010). Glutamate, excitatory amino acid transporters, Xc-antiporter, glutamine synthetase, and *γ*-glutamyltranspeptidase in human corneal epithelium. *Current Eye Research*.

[67] Li B, Lee MS, Lee RSY, Donaldson PJ, Lim JC (2012). Characterization of glutathione uptake, synthesis, and efflux pathways in the epithelium and endothelium of the rat cornea. *Cornea*.

[68] Lewerenz J, Klein M, Methner A (2006). Cooperative action of glutamate transporters and cystine/glutamate antiporter system Xc-protects from oxidative glutamate toxicity. *Journal of Neurochemistry*.

[69] Storck T, Schulte S, Hofmann K, Stoffel W (1992). Structure, expression, and functional analysis of a Na^+^-dependent glutamate/aspartate transporter from rat brain. *Proceedings of the National Academy of Sciences of the United States of America*.

[70] Ballatori N, Krance SM, Marchan R, Hammond CL (2009). Plasma membrane glutathione transporters and their roles in cell physiology and pathophysiology. *Molecular Aspects of Medicine*.

[71] Mahagita C, Grassl SM, Piyachaturawat P, Ballatori N (2007). Human organic anion transporter 1B1 and 1B3 function as bidirectional carriers and do not mediate GSH-bile acid cotransport. *The American Journal of Physiology—Gastrointestinal and Liver Physiology*.

[72] Li L, Meier PJ, Ballatori N (2000). Oatp2 mediates bidirectional organic solute transport: a role for intracellular glutathione. *Molecular Pharmacology*.

[73] Lash LH (2011). Renal membrane transport of glutathione in toxicology and disease. *Veterinary Pathology*.

[74] Aebi H, Flohé L (1974). *Glutathione: Proceedings of the 16th Conference of the German Society of Biological Chemistry, Tübingen, March 1973*.

[75] Giblin FJ, Chakrapani B, Reddy VN (1976). Glutathione and lens epithelial function. *Investigative Ophthalmology*.

[76] Reddy VN (1971). Metabolism of glutathione in the lens. *Experimental Eye Research*.

[77] Lim J, Lam YC, Kistler J, Donaldson PJ (2005). Molecular characterization of the cystine/glutamate exchanger and the excitatory amino acid transporters in the rat lens. *Investigative Ophthalmology and Visual Science*.

[78] Lim J, Lorentzen KA, Kistler J, Donaldson PJ (2006). Molecular identification and characterisation of the glycine transporter (GLYT1) and the glutamine/glutamate transporter (ASCT2) in the rat lens. *Experimental Eye Research*.

[79] Li B, Li L, Donaldson PJ, Lim JC (2010). Dynamic regulation of GSH synthesis and uptake pathways in the rat lens epithelium. *Experimental Eye Research*.

[80] Kahn MG, Giblin FJ, Epstein DL (1983). Glutathione in calf trabecular meshwork and its relation to aqueous humor outflow facility. *Investigative Ophthalmology and Visual Science*.

[81] Pattabiraman PP, Pecen PE, Rao PV (2013). MRP4-mediated regulation of intracellular cAMP and cGMP levels in trabecular meshwork cells and homeostasis of intraocular pressure. *Investigative Ophthalmology and Visual Science*.

[82] Do CW, Civan MM (2004). Basis of chloride transport in ciliary epithelium. *Journal of Membrane Biology*.

[83] Chu T-C, Candia OA (1988). Active transport of ascorbate across the isolated rabbit ciliary epithelium. *Investigative Ophthalmology and Visual Science*.

[84] Helbig H, Korbmacher C, Wiederholt M (1990). Mechanism of ascorbic acid transport in the aqueous humor. *Fortschritte der Ophthalmologie*.

[85] Takata K, Kasahara T, Kasahara M, Ezaki O, Hirano H (1991). Ultracytochemical localization of the erythrocyte/HepG2-type glucose transporter (GLUT1) in the ciliary body and iris of the rat eye. *Investigative Ophthalmology and Visual Science*.

[86] Socci RR, Delamere NA (1988). Characteristics of ascorbate transport in the rabbit iris-ciliary body. *Experimental Eye Research*.

[87] Ng M, Susan SR, Shichi H (1988). Bovine non-pigmented and pigmented ciliary epithelial cells in culture: comparison of catalase, superoxide dismutase and glutathione peroxidase activities. *Experimental Eye Research*.

[88] Ng MC, Shichi H (1986). Purification and properties of glutathione reductases from bovine ciliary body. *Experimental Eye Research*.

[89] Shichi H (1990). Glutathione-dependent detoxification of peroxide in bovine ciliary body. *Experimental Eye Research*.

[90] Singh AK, Shichi H (1998). A novel glutathione peroxidase in bovine eye: sequence analysis, mRNA level, and translation. *The Journal of Biological Chemistry*.

[91] Rao NA, Thaete LG, Delmage JM, Sevanian A (1985). Superoxide dismutase in ocular structures. *Investigative Ophthalmology and Visual Science*.

[92] Bhuyan KC, Bhuyan DK (1978). Superoxide dismutase of the eye. Relative functions of superoxide dismutase and catalase in protecting the ocular lens from oxidative damage. *Biochimica et Biophysica Acta*.

[93] Martin-Alonso JM, Ghosh S, Coca-Prados M (1993). Cloning of the bovine plasma selenium-dependent glutathione peroxidase (GP) cDNA from the ocular ciliary epithelium: expression of the plasma and cellular forms within the mammalian eye. *Journal of Biochemistry*.

[94] Hu RG, Lim JC, Kalloniatis M, Donaldson PJ (2011). Cellular localization of glutamate and glutamine metabolism and transport pathways in the rat ciliary epithelium. *Investigative Ophthalmology and Visual Science*.

[95] Li B, Umapathy A, Tran LU, Donaldson PJ, Lim JC (2013). Molecular identification and cellular localisation of GSH synthesis, uptake, efflux and degradation pathways in the rat ciliary body. *Histochemistry and Cell Biology*.

[96] Gao B, Huber RD, Wenzel A (2005). Localization of organic anion transporting polypeptides in the rat and human ciliary body epithelium. *Experimental Eye Research*.

[97] Aydin B, Yagci R, Yilmaz FM (2009). Prevention of selenite-induced cataractogenesis by N-acetylcysteine in rats. *Current Eye Research*.

[98] Carey JW, Pinarci EY, Penugonda S, Karacal H, Ercal N (2011). In vivo inhibition of l-buthionine-(S,R)-sulfoximine-induced cataracts by a novel antioxidant, N-acetylcysteine amide. *Free Radical Biology and Medicine*.

[99] Ohtsu A, Kitahara S, Fujii K (1991). Anticataractogenic property of *γ*-glutamylcysteine ethyl ester in an animal model of cataract. *Ophthalmic Research*.

[100] Mårtensson J, Steinherz R, Jain A, Meister A (1989). Glutathione ester prevents buthionine sulfoximine-induced cataracts and lens epithelial cell damage. *Proceedings of the National Academy of Sciences of the United States of America*.

[101] Rathbun WB, Holleschau AM, Cohen JF, Nagasawa HT (1996). Prevention of acetaminophen- and naphthalene-induced cataract and glutathione loss by CySSME. *Investigative Ophthalmology and Visual Science*.

[102] Rathbun WB, Nagasawa HT, Killen CE (1996). Prevention of naphthalene-induced cataract and hepatic glutathione loss by the L-cysteine prodrugs, MTCA and PTCA. *Experimental Eye Research*.

[103] Lou MF (2003). Redox regulation in the lens. *Progress in Retinal and Eye Research*.

[104] Brennan L, McGreal R, Kantorow M (2012). Oxidative stress defense and repair systems of the ocular lens. *Frontiers in Bioscience*.

[105] Yan H, Harding JJ, Xing K, Lou MF (2007). Revival of glutathione reductase in human cataractous and clear lens extracts by thioredoxin and thioredoxin reductase, in conjunction with *α*-crystallin or thioltransferase. *Current Eye Research*.

